# Non-homologous end joining induced alterations in DNA methylation: A source of permanent epigenetic change

**DOI:** 10.18632/oncotarget.16122

**Published:** 2017-03-11

**Authors:** Brittany Allen, Antonio Pezone, Antonio Porcellini, Mark T. Muller, Michal M. Masternak

**Affiliations:** ^1^ College of Medicine, Burnett School of Biomedical Sciences, University of Central Florida, Orlando, FL, USA; ^2^ Dipartimento di Medicina Molecolare e Biotecnologie Mediche, Istituto di Endocrinologia ed Oncologia Sperimentale del C.N.R., Università Federico II, Napoli, Italy; ^3^ Dipartimento di Biologia, Università Federico II, Napoli, Italy; ^4^ Epigenetics Division, TopoGEN, Inc., Buena Vista, CO, USA; ^5^ Department of Head and Neck Surgery, The Greater Poland Cancer Centre, Poznan, Poland, Europe

**Keywords:** DNA damage, NHEJ repair, DNA repair, DNA methylation

## Abstract

In addition to genetic mutations, epigenetic revision plays a major role in the development and progression of cancer; specifically, inappropriate DNA methylation or demethylation of CpG residues may alter the expression of genes that promote tumorigenesis. We hypothesize that DNA repair, specifically the repair of DNA double strand breaks (DSB) by Non-Homologous End Joining (NHEJ) may play a role in this process. Using a GFP reporter system inserted into the genome of HeLa cells, we are able to induce targeted DNA damage that enables the cells, after successfully undergoing NHEJ repair, to express WT GFP. These GFP+ cells were segregated into two expression classes, one with robust expression (Bright) and the other with reduced expression (Dim). Using a DNA hypomethylating drug (AzadC) we demonstrated that the different GFP expression levels was due to differential methylation statuses of CpGs in regions on either side of the break site. Deep sequencing analysis of this area in sorted Bright and Dim populations revealed a collection of different epi-alleles that display patterns of DNA methylation following repair by NHEJ. These patterns differ between Bright and Dim cells which are hypo- and hypermethylated, respectively, and between the post-repair populations and the original, uncut cells. These data suggest that NHEJ repair facilitates a rewrite of the methylation landscape in repaired genes, elucidating a potential source for the altered methylation patterns seen in cancer cells, and understanding the mechanism by which this occurs could provide new therapeutic targets for preventing this process from contributing to tumorigenesis.

## INTRODUCTION

DNA may be methylated on cytosine residues of CpG islands by the catalytic activity of DNA methyltransferases (DNMTs). DNA methylation may be classified as either invariant and stable (sex-specific imprinting) or metastable. In somatic cells, DNA methylation is metastable and changes with age [1], diet [2, 3], environment [4], disease [5–8], or other external or intrinsic events [9]. In this work, we are examining somatic, metastable DNA methylation. This epigenetic modification is usually associated with gene silencing [10, 11] as it interferes with transcription machinery and is recognized by proteins that recruit histone modifiers to condense chromatin, an action which blocks the accessibility of transcription machinery to the affected genes [12–14]. Under normal conditions, epigenetic modifications serve to modulate gene expression during embryonic development [15] and genomic imprinting [16], differentiation [17], or in response to stimuli [18]; however unscheduled changes can cause inappropriate gene silencing of tumor suppressor genes or activation of oncogenes, a phenomenon that is seen in various types of cancer cells [10, 19–21].

In order to pass the G2 checkpoint and enter mitosis, cells must repair any DNA damages that have occurred. Double stranded breaks in DNA (DSBs), in which breaks occur in both strands of the DNA double helix in close proximity to one another, are the most dangerous damage for a cell [22, 23]. These breaks can be caused by both exogenous and endogenous sources including reactive oxygen species, ionizing radiation [24], replication fork collapse [22, 25], or the faulty action of nuclear enzymes such as topoisomerase II [26, 27]. Regardless of origin, DSBs are fatal to the cell if not repaired. When faced with such a damage, the cell must repair the damage in order to survive or continue dividing [22]. Double strand breaks are repaired by one of two pathways in Eukaryotic cells [28]. During S and G2 phases of the cell cycle, when DNA has been replicated and exists in pairs of sister chromatids, the cell is able to fix the breaks with the high fidelity process of Homology Dependent Repair (HDR) [29, 30]. This process involves resection of one strand of a broken end to produce a single stranded overhang that can invade the helix of the sister chromatid. Polymerase then uses the sister chromatid to fill in sequence on the broken ends and the strands are resolved to separate, complete sister chromatids [26, 31]. During the rest of the cell cycle, or in non-dividing cells, no identical sequence template is available to allow HDR to proceed, so the cell turns to the faster but more error prone process of Non-Homologous End Joining (NHEJ) [32, 33]. This process proceeds through recognition and binding of the broken ends by Ku 70-80 proteins and DNA-PKcs [34]. Together, this complex has a role similar to that of Proliferating Cell Nuclear Antigen (PCNA) during replication as it acts as a docking platform for other proteins. During repair, these other proteins are nuclease, polymerase, and ligase complexes needed to process the repair. The DNA-PKcs complex with Artemis has 3’ and 5’ endonuclease as well as 5’ exonuclease activity, allowing it to process a diverse array of damaged DNA ends. Polymerases μ and λ are also able to interact with the complex, allowing flexible and template-independent synthesis. The processing of DNA ends during NHEJ is not fully understood and is not the same for each break; even identical breaks in the same location show variation in end processing [34]. Blunted DNA ends are subsequently ligated through the action of XLF:XRCC4:DNA ligase IV complex. Although the immediate threat to the cell is averted by repair of the DSB, repair by NHEJ often results in deletions or frame shifts in the repaired area as a result of end processing. This process is a major source of DNA mutation in arrested cells [35]. Despite its limitations, the quick kinetics and ability to repair without a template make NHEJ the repair pathway of choice in cells outside of S and G2 and in non-dividing cells. It is the predominant DSB repair pathway in animal cells since it occurs throughout the cell cycle [32, 33].

Studies of HDR have determined that, following repair, some cells exhibit robust expression of the repaired gene while others show low expression levels. It was determined that these expression classes arise as a result of epigenetic reprogramming, more specifically, altered CpG methylation at the repair site [36–38]. In addition, specific DNA methyltransferases have been found to localize at DNA repair sites [36]. During S phase, DNMT1 methylates hemimethylated DNA during replication, copying the methylation profile of the parental DNA strand to the daughter strand, a process which is also ongoing when DNA methylation is altered post-HDR [36, 39]. Thus, DNA methylation exists in a triad of dynamic, interworking processes along with DNA replication and HDR.

The mechanism for DNMT-mediated methylation at repair sites following NHEJ, which occurs independently of DNA replication, is less well understood despite being a prominent DNA repair pathway in animal cells. We address this topic in the current paper. Specifically, in this work, we report the following observations. First, NHEJ repair pathway attended by DNA methylation revision in somatic human cells. Second, specific methylation sites map on the repaired gene to sites that are distinctly different from those seen in the HDR pathway [36]. Third, we show that epigenetic revisions driven by NHEJ are stably inherited. In this work, we used a neutral gene to report alterations in DNA methylation to ensure that selective pressure post repair would not influence our ability to track the NHEJ descendants. Collectively, the data supports the notion that the prominent DNA repair pathway in animal cells is a source of genetic diversity but also a source of epigenetic (or gene expression) change in cases where a wild type allele is recovered post-NHEJ.

## RESULTS

### A neutral reporter system for analysis of epigenetic revision during NHEJ

**Figure 1:**
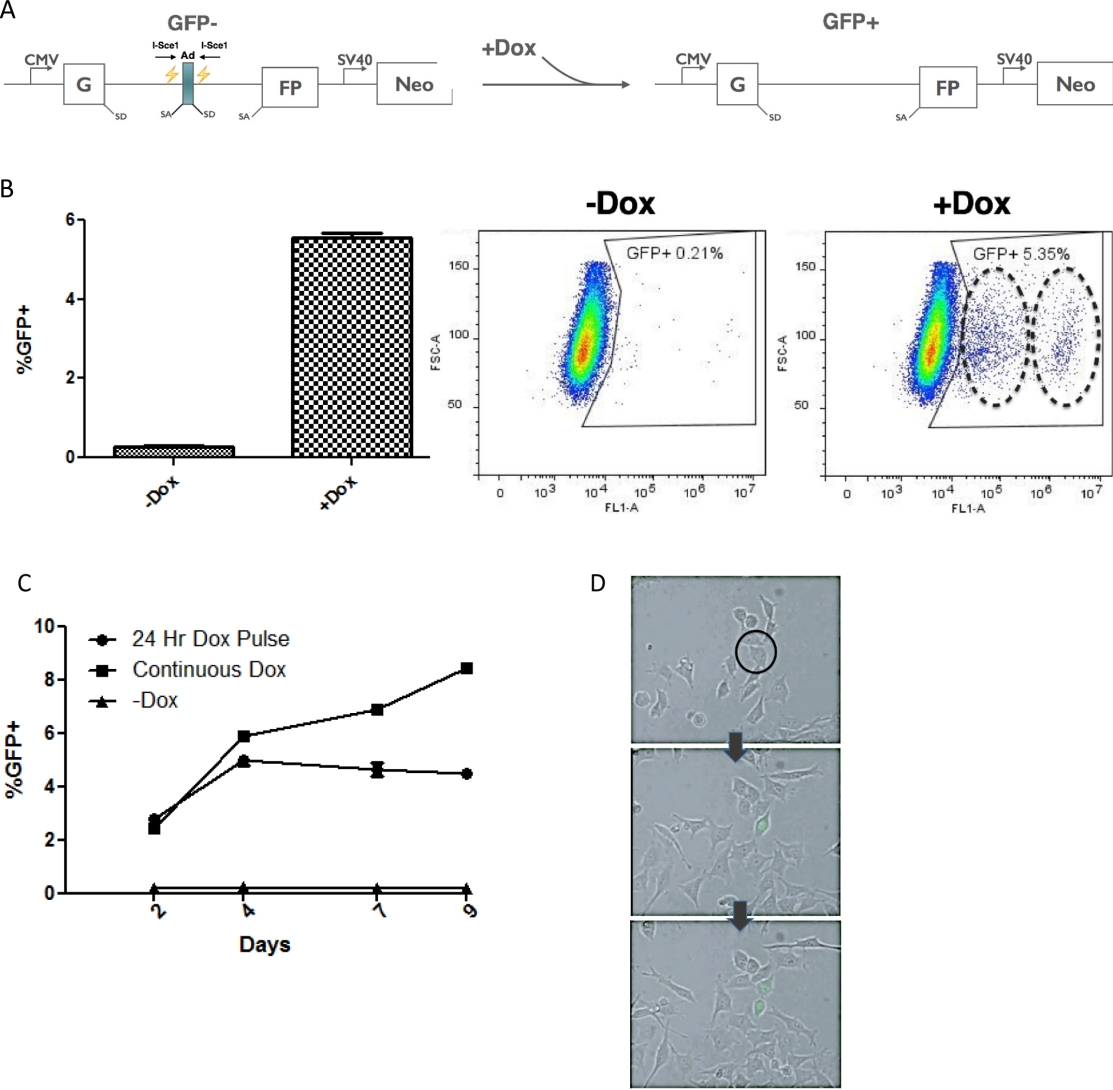
Doxycycline inducible construct uses GFP as a reporter for NHEJ **(A)** Reporter construct integrated into the genome of the IHN20.22 HeLa cell line. The NHEJ reporter GFP gene contains a Pem1 intron interrupted by an adenoviral exon. Two I-Sce1 restriction sites allow the homing endonuclease to cut the DNA and excise the adenoviral exon to produce wild-type GFP following repair by NHEJ. **(B)** Generation of GFP positive cells following repair. Cells were induced with dox for 24 hours and then the percentage of the population expressing GFP was analyzed using FACS. The circles on the “+Dox” plot indicate two separate GFP positive cell populations with differing expression levels of GFP **(C)** Time course analysis. The percentage of GFP positive cells was analyzed by FACS over the course of 9 days, either following a 24-hour induction or with continuous exposure to doxycycline. The uncut cell line with no dox exposure was also analyzed to asses basal levels of GFP expression. **(D)** The onset of WT GFP expression in a single cell during the 72 hours following a 24 hour Dox induction was observed using live-cell imaging. The arrows indicate time progression and the black circle indicates the GFP negative cell that is GFP positive in subsequent images.

In order to study the NHEJ repair pathway, we have generated a HeLa cell line containing a GFP based reporter construct [40, 41] (Figure 1A). The construct contains a CMV driven GFP gene that was interrupted by a rodent Pem1 intron. Within this intron, an adenoviral exon was added that is flanked by two restriction sites for the megaendonuclease, I-Sce1. There are no I-Sce1 sites in the human genome; Thus DSB are target to these twin sites. The presence of the viral exon disrupts the gene, and as a consequence, the cells are GFP negative; however when I-SceI is introduced to the cells, the adenoviral exon is excised by two DSBs and repaired by NHEJ. Since removal of the exon allows the construct to generate WT GFP after splicing, cells have effectively undergone NHEJ repair and some sub-fraction of the cells will be GFP positive (since NHEJ is error prone, we cannot score mutant alleles with this assay based on GFP expression). In addition, there is no homologous DNA template for the repair event; therefore, this is a dedicated NHEJ repair process and HDR cannot proceed under these conditions. To improve accuracy and penetrance of the system, the gene for I-SceI gene has been placed in these HeLa cells under control of a Tet-On promoter, thus, the system is doxycycline inducible.

### A segregation of expression classes following repair by NHEJ

The NHEJ reporter system was tested by adding Doxycycline (Dox) to the media of the IHN20.22 HeLa cells. We note that other clones tested behaved similarly; however some clones displayed higher backgrounds, probably due to leaky I-Sce1 expression in the absence of Dox. IHN20.22 was selected since this clone exhibited low levels of GFP positive cells in the absence of Dox. I-Sce1 induction was quite robust in IHN20.22 cells and within a few hours after Dox addition, Western blots showed the presence of prominent amounts of I-Sce1 protein (data not shown). At 24 hours post-Dox, GFP expressing cells could readily be seen by live imaging or fluorescent microscopy. GFP levels could also be measured using flow cytometry (Figure 1B). The percentage of GFP positive cells steadily increases over time with continuous exposure to Doxycycline, while in negative controls (no Dox) the percentage of GFP positive cells stays well under 1% which we attribute to leakiness of the Tet-On promoter, as noted. After a 24 hour pulse with Dox, the percentage increases and then peaks at about 5-7% after four days (Figure 1C). The emergence of GFP positive cells can be observed via live imaging during the 72 hours following induction with Doxycycline (Figure 1D) as a GFP negative cell (circle, top image) undergoes NHEJ repair and begins to express WT GFP as time progresses to the second image. Since the post-repair cell now contains a WT GFP gene, division produces two GFP positive daughter cells in the last image. By limiting the expression of I-Sce1 to 24 hours, the pulse-chase experimental schematic allows for focused investigation of the processes that occur following a DSB without the added variables of continuous DSB induction and continuous Dox treatment. Note that the scatter plots in Figure 1C appear to contain dual populations of GFP positive cells (dashed rings). The two populations, which appear to differ in total GFP expression levels were more clearly observable after Dox induction with I-Sce1; however in the negative controls, this heterogeneity was observed. This was examined in more detail in order to understand the underlying basis for this observation.

### GFP Expression is heterogeneous in repaired cell populations following NHEJ

**Figure 2:**
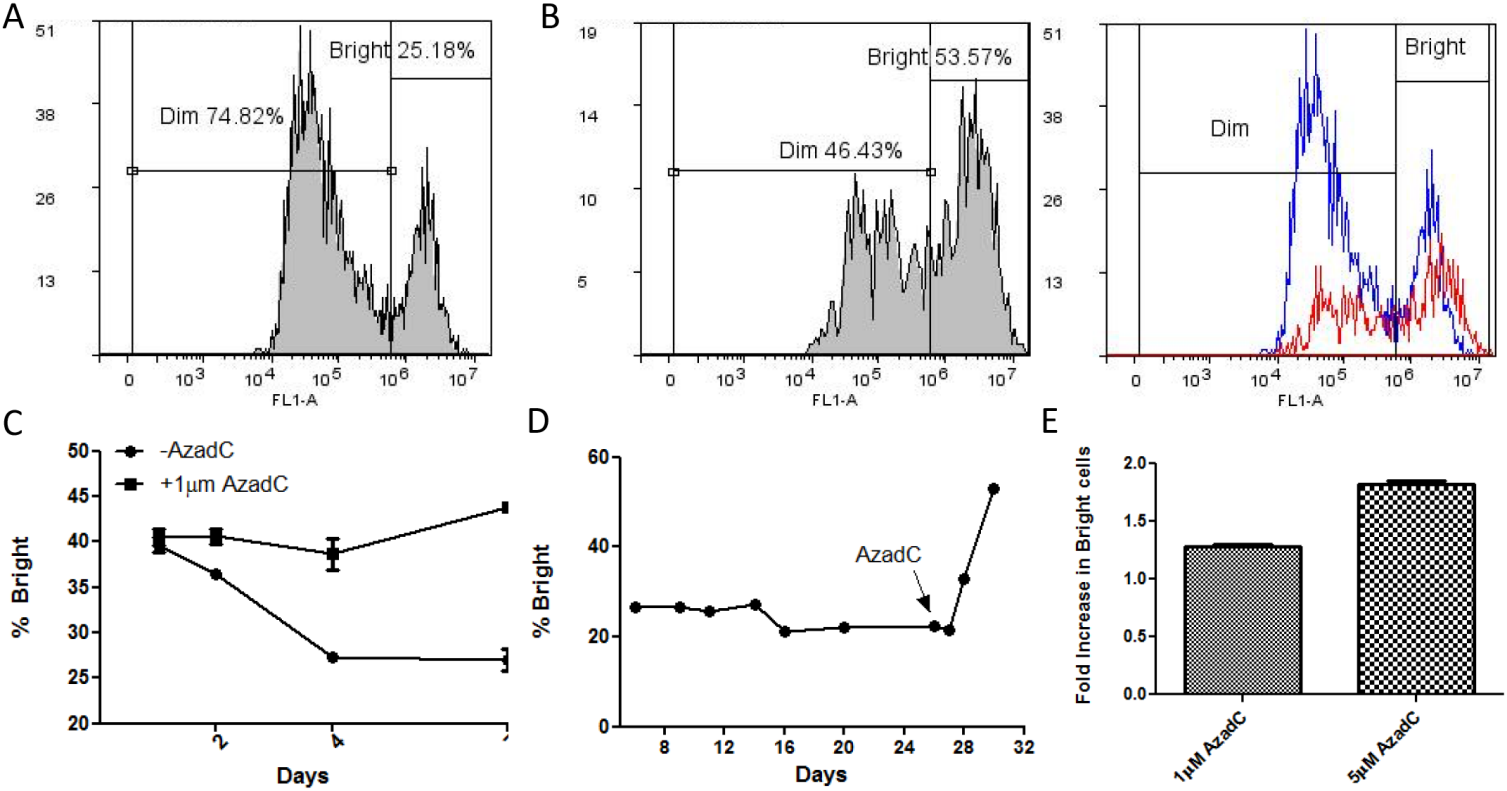
The effect of induced hypomethylation on the distribution of cells in Bright and Dim GFP expression classes **(A)** Histogram of GFP+ cells after induction with dox with gating for Bright and Dim populations. **(B)** Hypomethylation by 5’Aza-2’-deoxycitidine. IHN20.22 cells were induced with dox for 24 hours and then treated with a daily dose of 1μM AzadC for 48 hours. The percentage of cells with high (Bright) and low (Dim) GFP expression with and without AzadC treatment were quantified using FACS histograms of GFP positive cells. The second graph is an overlay of the histogram with (red) and without (blue) treatment with AzadC. **(C)** Characterization of the effect of hypomethylation by AzadC on the GFP expression level in post-repair cells. The first graph compares the GFP expression level over the course of 7 days with and without AzadC treatment. Cells were induced with dox for 24 hours and then given a daily dose of 1μM AzadC. The percentage of Bright cells was measured using FACS on days 1, 2, 4 and 7 following initiation of AzadC treatments. **(D)** The distribution of cells in each expression class was observed in the days following NHEJ repair. As indicated by the arrow on the last graph, the cells were treated with 5μM AzadC on day 25 and then analyzed by FACS on day 26, 27, 28, and 30. **(E)** Comparison of the effect of AzadC at different concentrations. Cells were induced with dox for 24 hours and then given a daily dose of the indicated concentration of AzadC for 2 days. After a 48-hour recovery, the percentage of Bright cells was determined using FACS. The fold increase in the percentage of bright cells is shown.

The histogram of GFP positive cells reveals the emergence of two expression classes with differing GFP intensities. One class expresses GFP robustly while the other maintains lower expression levels. We refer to these populations as Bright and Dim, respectively (Figure 2A). To determine if the differing expression classes may be a result of DNA methylation, we tested whether the expression classes were altered by the DNA hypomethylating drug, 5’-Aza-2’-Deoxycytidine (AzadC). This drug acts by inducing a stable, covalent complex between the methyl's and DNA [36, 42–44]. The result is hypomethylation of the genome due to the sequestering of DNMTs that are covalently bound to DNA. This prevents further methyltransferase action and effectively inhibits the overall DNA methylation of the cell. Based on this known mechanism, multiple cell divisions are required in order to observe genome-wide hypomethylation, which is manifested in the daughter cell population.

When IHN20.22 cells are induced with Dox and then treated with AzadC, there is an obvious shift of cells from the low expressing Dim population to the high expressing Bright population (Figure 2B). The conversion of ‘dim’ cells to ‘bright’ cells by the hypomethylating drug suggests that the repaired GFP gene in the low expressing pool is a direct result of DNA methylation either during or soon after NHEJ repair. In the absence of AzadC, the Bright population of cells initially decreases over 4 days then appears to stabilize (Figure 2C, -AzadC). In contrast, cells treated with AzadC displayed a clearly different trend (Figure 2C, -AzadC). The drug reverses the loss in Bright cells, probably due to the ongoing conversion of Dim cells into the Bright pool. In either case, a few days after the damage/repair event, the proportions of cells in Bright and Dim cell populations remain fairly stable as cells are passaged (Figure 2C); however, addition of AzadC to cells that are 24 days post-repair results in a sharp increase in the percentage of high expressing cells (Figure 2D), supporting the notion that the expression difference is likely due to post-repair methylation as opposed to an off target drug interaction during the repair process. Further, the extent to which AzadC causes the shift from low to high GFP expression occurs is dose dependent (Figure 2E).

**Figure 3:**
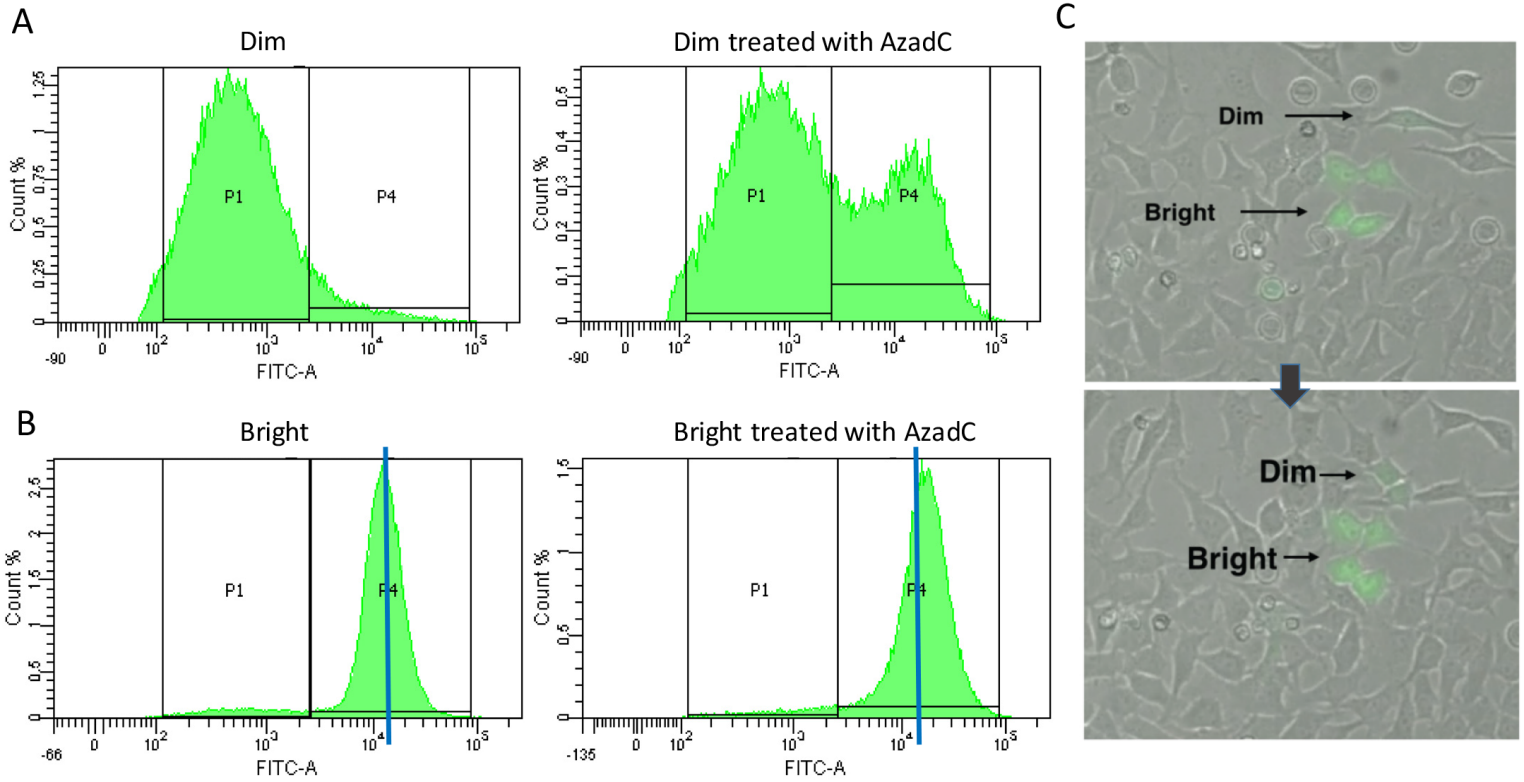
(A, B) Dim (A) and Bright (B) populations were sorted using FACS and then the sorted populations were treated with a daily dose of AzadC for 48 hours The level of GFP expression was measured using FACS. The blue line in **(B)** indicates the median of the Bright peak. **(C)** Live cell imaging was used to observe the origination and propagation of cells with Dim and Bright expression of GFP during the 72 hours following a 24 hour dox induction. The arrow indicates the progression of time

Since it appears that the expression classes eventually become stable, presumably due to stable methylation marks (which are heritable) we next attempted to isolate pure populations of Dim and Bright cells to make the analysis more tractable. Before treatment, sorted Dim cells appear as a relatively homogenous population in a uni-modal distribution (Figure 3A) with relatively low levels of GFP expression (labeled as the “P1” pool). Note that while the distribution is broad, it is nonetheless uniform with very low levels of bright GFP expressing cells (P4). Following AzadC treatment of the Dim cell pool, a new population emerges in the high expression range (Figure 3A right panel “P4” pool). As with sorted Dim cells, sorted Bright cells also show a single peak of GFP expression (Figure 3B); however these cells are far more homogeneous (compare 3A, B). Since the Bright cell pool also shifts perceptibly to the right in the presence of AzadC (Figure 3B, right panel, P4), we conclude that the bright pool contains cells with some degree of DNA methylation (which is removed by AzadC) (Figure 3B). This experiment clearly evidenced the conversion of Dim cells to Bright cells by a DNA hypomethylating drug. Live imaging reveals that the information regulating the GFP expression level of post-repair cells is passed from parent to daughter cells as the cells divide (Figure 3C, Supplementary Movie 1). The cause of the silencing is heritable but reversible, pointing once more to DNA methylation as the source of the gene expression variation.

**Figure 4:**
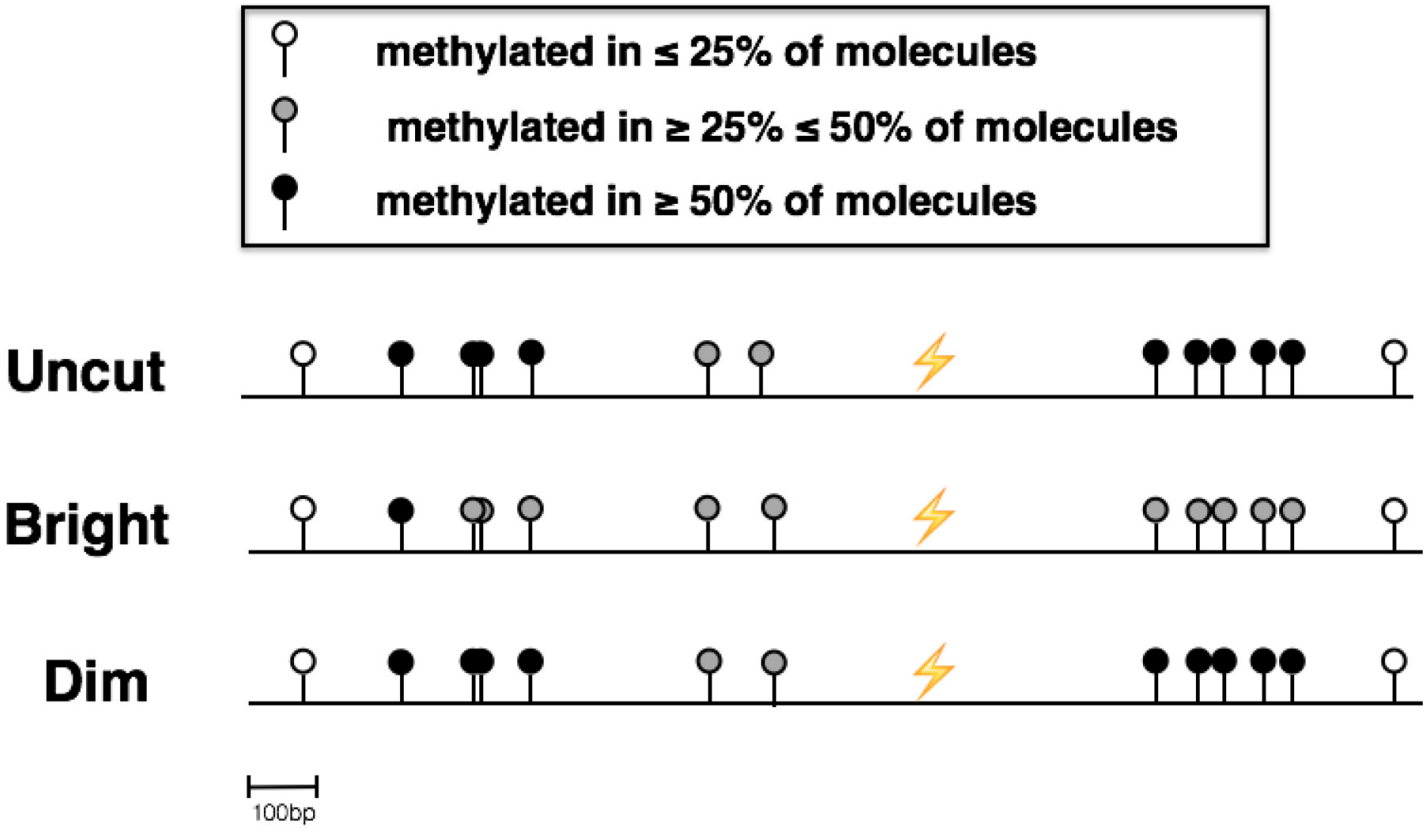
Bisulfite Sequencing of sorted Dim and Bright cells Next-Generation Sequencing of bisulfite converted DNA was used to determine the methylation patterns of the area around the site of repair in sorted Dim and Bright cell populations as well as uncut IHN20.22 cells

The availability of relatively pure populations of Dim and Bright cells makes it possible to use bisulfite DNA sequencing to interrogate methylation sites before and after NHEJ. Thus, in addition to validating the presence of epi-alleles in Dims and Brights, bisulfite DNA sequencing of the post-repair populations makes it possible to map these epi-alleles relative to the I-Sce1 cleavage and repair site. Bisulfite sequencing confirmed that the GFP gene is hypomethylated in sorted Bright cells and hypermethylated in sorted Dim cells. Specifically, changes occur in regions both up and downstream of the break site (Figure 4). Interestingly, the methylation status of CpGs in the region directly flanking the break site is not affected by the process. This suggests a coordination of methylation and repair proteins to affect methylation patterns in specific areas around the site of DNA damage.

### Different DNA methylation patterns mark RECOMBINANT from UNCUT cells

The data so far demonstrate that NHEJ is spinning out new epi-alleles that are either over-written or completely revised from the parental (uncut) reporter DNA. Moreover, the expressability of the repair products correlates with the percent of DNA methylation in sorted Dim and Bright cells. The pyrosequencing analysis done thus far evaluates the average methylation levels for a single CpG site derived from physically different molecules; however it does not consider the relationship between the different methylated cytosines present on the same molecule (epialleles). For this reason, we have analyzed the composition of methylated population (heterogeneity), by counting the number of different epialleles in the sample (haplotypes) obtained from deep sequencing analysis of the amplicons.

**Figure 5:**
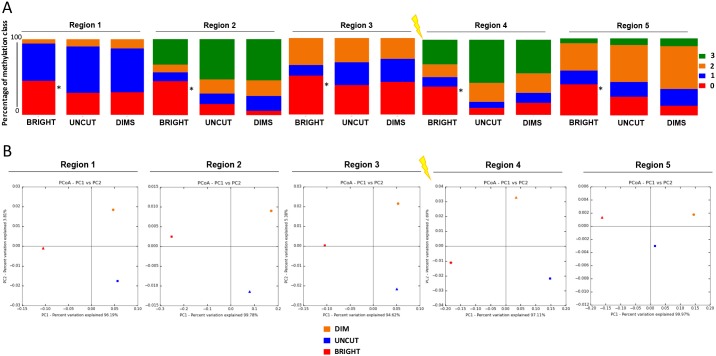
Qualitative DNA methylation profiles discriminate BRIGHT molecules from DIMS and UNCUT molecules Epialleles profiles obtained from the analysis of the methylation of each amplicons were subjected to alpha and beta diversity (Qiime). **(A)** Profile composition of relative sample (BRIGHT, DIMS and UNCUT) grouped by number of methylated CpGs for the five regions adjacent to the DSB. “0 (red color = un-methylated)”, “1 (blue = mono-methylated)”, “2 (orange = di-methylated)” and “3 (green = tri-methylated)” represent the percent to class of methylation. **(B)** Principal coordinate analysis of BRIGHT, DIMS and UNCUT. In the X and Y axes are represented, respectively, the first and the second components (PC1 and PC2) with the amount of variance in the samples explained by these components, included in brackets. The first principle component represents the highest variance, and the total variance of the samples is the cumulative sum of that described by each of the axes.

**Figure 6:**
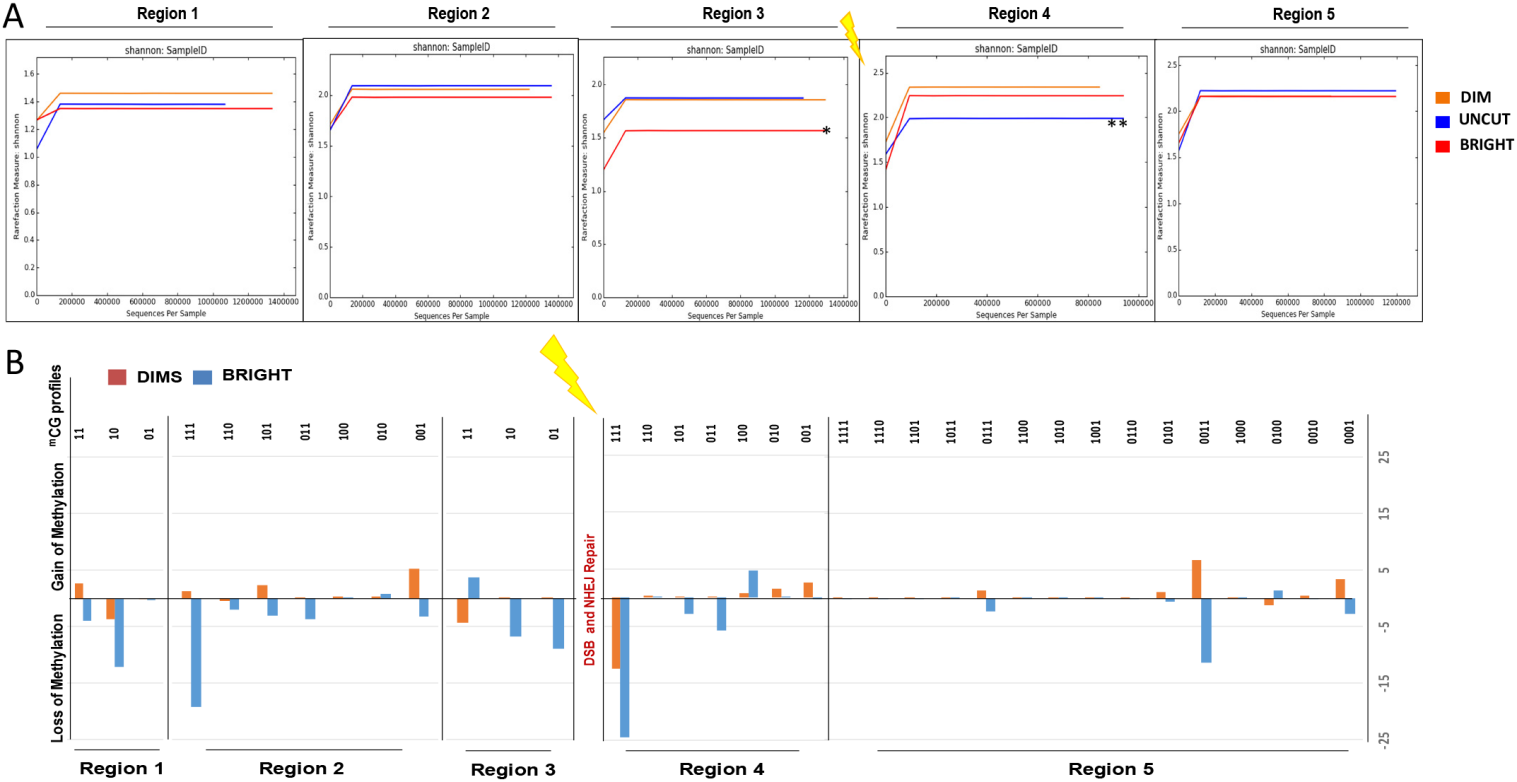
Regions adjacent to DSB give discriminate REC from UNCUT and BRIGHT from DIMS Epiallelesprofiles obtained from the analysis of the methylation of each amplicons were subjected to alpha and beta diversity (Qiime). **(A)** Shannon diversity index between BRIGHT, DIMS and UNCUT. In the X and Y axes are represented, respectively, “number of sequences for sample” and “Rarefaction measure (species richness)” for Shannon Index. **(B)** Gain or loss of methylation in BRIGHT and DIMS compared to the UNCUT.

The DSB region was divided into 5 segments (3 upstream and 2 downstream regions from the DSB site, see top diagram in Figure 5). Deep quantitative (Taxa, PCoA and Shannon Index) and qualitative (methylation profiles) analysis of amplicons with the same end was carried out. The results show that recombinant cells (both Dims and Brights) and uncut parental cells have the same types of methylated species (un-methylated, mono, bi and tri-methylated) but with different compositions (Figure 5A, Supplementary Table 1). In fact, the Bright cells appear to be rich in unmethylated species compared to Dims and Uncut in each region, confirming that high levels of GFP expression in this population are largely unmethylated (marked by an asterisk in Figure 5A). Moreover, Principal Coordinates Analysis reveals that Dim and Uncut parental DNAs have a smaller euclidean distance compared to Bright (variation 96% *vs* 4%), highlighting a common origin for Recombinant (ie, NHEJ repaired) cells, followed by a de-methylation event of the Bright cells (Figure 5B). We also analyzed the diversity index (Shannon Index) in order to evaluate the evenness of the species. Except for the region 1 (where Bright and Uncut are much more similar to the Dims), we observe that 2 upstream regions of DSB site (Region 2 - 3) show a gradual increase in similarity between Dims and Uncut relative to the Bright cells (single asterisk*), while region 4 (downstream region of DSB site) shows a high similarity between the Recombinants (Dims plus Brights) compared to the Uncut (double asterisk **), which is lost in the region 5 (Figure 6A).

Finally, we have performed a deep qualitative analysis to identify the differences between Recombinant and Uncut (parental) cells in terms of gain or loss of methylation. The differences between the percentage of Recombinant's epialleles compared to Uncut cells show that the Dim cells gain methylation marks in the region 1 (*di-methylated epialleles*), in the region 2 (*mono-, di- and tri-methylated epialleles*), and in the region 5 (*mono-, di- and tri-methylated epialleles*). In contrast the Bright cells lose methylated epialleles in these same regions. For example, in region 3, Bright cells appear to acquire new methylation (*di-methylated epialleles*) while DIMS lose methylation. Both Dims and Bright cells lose methylation (*tri-, di-methylated epialleles)* acquiring mono-methylated epialleles in the region 4. Knowing the different GFP expression between the two populations, we hypothesize that transcription may modify DNA methylation (or de-methylation) after DNA damage and NHEJ repair, remodeling the chromatin in units with different transcriptional efficiency (and in the process spinning out Dim and Bright cell populations) [38].

## DISCUSSION

### Epigenetic revision as a component of tumorigenesis

This study reveals a novel connection between two essential cellular processes that has a potential role in the development and progression of cancer. Cancerous cells arise in a multi-step process as a result of an upset in the balance of oncogenes and tumor suppressor genes that causes deregulation of normal epistasis. This imbalance may derive from genetic mutations that affect this balance; however changes in expression levels can also be caused by inappropriate DNA methylation marks. Moreover, the addition or removal of post-translational modifications to histones can affect the access of transcriptional machinery to particular genes by opening or closing the chromatin, causing gene activation or repression, respectively [11, 19, 45, 46]. Both hypermethylation and hypomethylation can contribute to tumorigenesis when they occur at inappropriate positions in the genome. DNA hypermethylation is generally associated with gene silencing while hypomethylated is associated with gene activation [19, 20]. Changes in the cellular DNA methylome resulting in hypermethylation of tumor suppressor genes including APC, BRCA1, E-cadherins, DAPK1, hMLH1, p15, Rb, MGMT, and p16INK4a have been documented in tumors from a variety of cancers including breast, colon, gastric, ovarian, lung, brain, ovarian, renal, kidney, prostate, thyroid, lymphoma, and leukemia [11, 19, 20]. In addition to silencing tumor suppressor genes, hypermethylation can cause down regulation of miRNAs, some of which have tumor suppressing activity [47–49]. For example, miR124a is frequently down regulated in several cancer types including colon, breast, and lung carcinomas as well as some leukemias and lymphomas. Because this miRNA is a negative regulator of CDK6, down regulation caused by hypermethylation results in increased levels of CDK6 which in turn facilitates inactivation of RB1 via phosphorylation [50] Since miRNAs have also been shown to regulate epigenetic processes through interaction with DNMTs and EZH2 complexes [51, 52], downregulation could potentially propagate DNA methylation changes in other areas of the genome. Hypomethylation has also been shown to contribute to tumorigenesis by over-activating oncogenes and contributing to genetic instability and structural changes by promoting an open chromatin state [20, 53].

Cancer cells coordinate genetic mutations and epigenetic revision in order to promote carcinogenesis. According to the two-hit hypothesis, a tumor suppressor gene must be deactivated in both alleles in order to cause cancer. HCT116 colon cancer cells were found to have genetic mutations in one allele of both CDKN2a and MLH1 and epigenetic silencing of the second allele, demonstrating the collaboration of genetic mutation and hypermethylation in achieving the second ‘hit’ to cause loss of heterozygosity and inactivation a tumor suppressor gene [20]. Both hypermethylation and hypomethylation can also contribute to cancer progression by promoting mutagenic processes. This is observed in the silencing of genes involved in DNA repair via hypermethylation, which results in defective repair pathways and increased DNA mutagenesis [45, 54]. In addition, hypomethylation can cause over expression of an oncogene due to the loss of genomic imprinting that results in the expression of both alleles instead of only one allele [55, 56], and hypomethylation of LINE retrotransposons facilitates insertion mutagenesis [57, 58].

Despite their involvement in so many types of cancer, the root cause of DNA miscues is largely unknown. Deep sequencing analysis of post-repair DNA revealed both loss and of methylation in areas up and downstream of the break site (Figure 6B) that is correlated with up- or downregulation, respectively. The mechanism described here provides evidence for alterations in methylation profiles as a result of NHEJ repair, a process which could explain the epigenetic revision that is characteristic of cancer cells. What is not clear is how universal and wide-spread NHEJ-mediated epigenetic revision paths are in a tissue context. In our model system, we used a gene that is not subject to selection; however it stands to reason that silencing or activating a positive or negative growth-promoting gene could produce cells that have a growth advantage relative to surrounding normal cells [11, 20, 59]. Growth promoting outcomes could clearly place the cell on a path toward a pre-cancerous condition. In contrast, silencing a pro-growth gene (oncogene) could result in the loss of a cell lineage. In either situation, tissue epistasis could be targeted with undesirable outcomes. What is intriguing is that even when an error-prone pathway such as NHEJ regenerates wild-type sequence, there is still a good chance for gene activation or silencing. Thus, mutating a tumor suppressor gene is functionally equivalent to epigenetic silencing the expression of the same gene, with the same dire consequences.

### A new pathway for discovery of novel epi-therapeutic targets

Changes in DNA methylation have been shown to accumulate throughout the genome with the progression of cancer [59–62] and have been shown to positively correlate with tumor stage [61]. Cancer cells have also been shown to acquire additional genetic mutations due to an increase in DSBs resulting from increase reactive oxygen species production, telomeric dysfunction, genomic instability, and replication errors [33, 63]. Although the increased DSB may give cancer cells a growth advantage by providing favorable mutations in key regulatory genes, it also makes them increasingly dependent on DSB repair in order to grow and proliferate at a high rate. If DNA methylation revisions occur during or soon after NHEJ, as demonstrated here, further DSB repair that occurs in rapidly dividing cancer cells may exacerbate the situation and could explain the accumulation of inappropriately methylated or demethylated genes that is characteristic of progressing cancer.

One important property of epigenetic alterations that makes them an idea therapeutic target is that they are reversible. This is in contrast to genetic mutations that are embedded in the DNA sequence. In fact, hypomethylating drugs including 5’Aza-2’-deoxycitidine (Decitabine), and 1-β-D-ribofuranosyl-2(1H)-pyrimidinone (Zebularine), have been shown to have positive effects when used to treated cancer cells. The FDA has approved the use of Decitabine for treating myelodysplastic syndrome and Zebularine for treating hematological malignancies [19, 46, 56, 62]. Studies have also shown that antisense and small interfering RNA (siRNA) targeting DNA methyltransferase mRNA can inhibit growth of colon and renal cell carcinoma cells [46, 56]. The association of methylation revisions with tumor stage could enable their use as a cancer marker to predict prognosis of developing cancer and to predict responsiveness to specific therapeutics [56]. Further, although methylation aberrations tend to accumulate with malignant progression, they have also been shown to be present in the early stages of pre-malignancy [20, 62, 64]. This could make them candidates for use as a diagnostic tool for early detection of cancer cells [62, 64]. Further, exposing the mechanism behind the overlap of these key processes could provide new targets for therapeutics to interfere with the progressive gain of epigenetic miscues by this mechanism and prevent tumor progression.

### Intragenic DNA methylation and gene silencing

While methylation based gene silencing is typically associated with promotor CpG islands, studies have also shown that intragenic methylation may affect gene expression as well. Intragenic methylation in plant cells was shown to increase in more highly transcribed genes, presumably in an effort to prevent aberrant transcription at other nearby sites that can result when transcription machinery disrupts chromatin structure. Inhibition of DNA methylation in these cells caused up regulation of genes that were methylated only in the body of the gene and not in their promoters [65]. Similar results were found in mammalian cells where intragenic methylation was shown to decrease gene expression and facilitate compaction of chromatin that is correlated with a reduced density of DNA polymerase II in the body of the gene. Further, another study concluded that a single methylated CpG in the intron of the PMP24 gene was sufficient to silence the gene. In addition to demonstrating the ability of intragenic methylation to silence genes, these studies suggests a connection between methylation and reduced elongation efficiency. This supports our findings that aberrant methylation in the body of the GFP gene causes silencing.

This also incites the idea that transcription may reshape the epigenetic landscape, a phenomenon that has already been demonstrated to occur following DNA repair by HDR [37]. It was found that this process is mediated by Base Excision Repair (BER)-mediated demethylation [38]. Principle Coordinates Analysis presented in Figure 5B revealed that Dim cells had a closer Euclidian distance to the original, uncut parental DNA than Bright cells. This data supports a model where the area surrounding the site of NHEJ is first methylated and then progressively demethylated over time with transcription, resulting in an array of epialleles with differing levels and locations of methylated CpGs. Future work will look further into this idea.

In summary, this study reveals a novel connection between two processes that have important roles in cancer development and progression, NHEJ and DNA methylation. This link provides a clue to one of the biggest unanswered question in this area of study: What causes the epigenetic revision that is pivotal to the multistep progression of cancer? While DNA damage and repair is stochastic, a damage event that results in epigenetic revision following repair and provides the cell with a growth advantage could direct the cell onto a path of tumorigenesis. This could explain why some genes exhibit inappropriate methylation patterns in some cancers but not others, as only genes that are beneficial to that particular tissue type would confer a selective advantage. Thus, methylation revision following DNA repair by NHEJ, the most predominant repair pathway in animal cells, fits the criterion to be the source of epigenetic abnormalities in cancer cells and could provide information for the development of new therapeutic strategies for preventing and stopping this process from contributing to tumorigenesis.

## MATERIALS AND METHODS

### Cell culture

The stable HeLa cell line was cultured in RPMI medium with L-glutamine and supplemented with 10% fetal bovine serum and 1% penicillin-streptomycin. Cells were grown in at 37°C at 5% CO_2_.

### Stable cell line

HeLa cells were transfected with a Tet-On inducible gene expression system (Clontech) and set to target the I-Sce1 gene according to the Tet-On system manual. After generation of a stable Tet-On cell line, the cells were transfected with the GFP reporter construct for NHEJ provided by the Gorbunova lab [40]. These cells were grown under selective pressure with Geneticin (G418) to generate the stable iHN20.22 cell line containing the reporter construct and a tetracycline inducible I-Sce1 gene.

### Tet on promoter activation

iHN20.22 cells were treated with 1μg/mL doxycycline for varying durations to determine the optimal induction time. It was determined that a 24 hour pulse of doxycycline was sufficient to induce the NHEJ system, so this was used in future experiments.

### FACS analysis

IHN20.22 cells were trypsinized and centrifuged at 1000xg for 5 minutes and then and resuspended in PBS at a density of 10^6^ cells/mL. Live cells were selected using a plot of SSC-A vs. FSC-A, and GFP positive cells were identified using a plot of FSC-A vs FL1A-A. The FL1-A histogram was then used to identify and gate the distinct populations of low and high expressing cells.

### Live cell imaging

Cells were seeded at low density in glass bottom culture dishes (MatTek) induced with Dox for 24 hours and then placed into an incubation chamber that is part of a Perkin Elmer UltraVIEW VoX 3D Live Cell Imaging System attached to a Zeiss Axio Observer Z1 inverted fluorescence microscope. Images were taken every 5 minutes for the next 48 hours and then analyzed using Volocity Imaging and Analysis software (Perkin Elmer).

### Drug treatments

IHN20.22 cells were plated at low confluence and induced with doxycycline for 24 hours. After 24 hours, media was removed and replaced with fresh media and cells were given a daily dose of 1μM 5-AzadC or 1% DMSO control for 48 hours (unless otherwise noted) and then were harvested for FACS analysis.

### Cell sorting

Dox induced IHN 20.22 cells were treated with dox for 24 hours and then grown under normal culture conditions until the GFP expression levels stabilized. Cells were then harvested and resuspended in median containing RPMI, 1% penicillin-streptomycin, 20mM Hepes buffer, and 2% FBS at a density of 3×10^6^ cells/mL. Cells were run through FACSAria flowcytometer and sorted by using the FSC-A and FITC-A plot to gate GFP positive cells and then using the histogram of FITC-A to select ‘dim’ and ‘bright’ populations. Two sorted populations (Bright and Dim) were collected in a tube containing media supplemented with 30% FBS and then transferred to culture dishes containing culture medium (RPMI supplemented with 10% FBS and 1% Penicillin-streptomycin) at 37°C at 5% CO_2_.

### Bisulfite converted DNA preparation

Genomic DNA was extracted from iHN20.22 cells (uncut) as well as the sorted bright and dim populations using Wizard genomic DNA purification kit” (Promega). 100ng of extracted DNA was used for bisulfite conversion using the Epijet Bisulfite Conversion Kit (Thermoscientific). Regions on either side of the repair site in bisulfite converted DNA were amplified using Phusion U Hot Start Polymerase (Thermoscientific) and the following 5 sets of primers (NHEJ1-5): NHEJ1-F, 5’-GTGATTATGGTTTTGTTTTTTTTTTTGGAATTGT -3’; NHEJ1-R, 5’-CTAACACTCCCTACTTAATAAAAACTCC-3’; NHEJ2-F, 5’-GGAGTTTTTATTAAGTAGGGAGTGTTAG-3’; NHEJ2-R, 5’-CCCCATAAAAACCCACAATATTTCAAATC-3’; NHEJ3-F,; 5’-AGGTTAGTTTGGGTTATATGAGAGTTTG-3’; NHEJ3-R, 5’-TTTCAAACTACCCCATATAACATCTAACC-3’; NHEJ4-F, 5’-TGGTAAGGGATTTTGTAGATTATTGGATTTAG-3’; NHEJ4-R, 5’-CTACTATACTCACCCATTATTCTAAAAACAC-3’; NHEJ5-F 5’-AGGTTGTATTTTATTTTTATAGTTAGGTTTGTTTAGG-3’; NHEJ5-R 5’-ATCTAAAAATACATTAAAAAATCCTCTTTCCCCTTC-3’; The 5 PCR fragments of each sample (uncut, sorted Dim, and sorted Bright) were purified using GENEjet PCR purification kit (Thermoscientific).

### Bisulfite sequencing

These samples were transferred to the core sequencing facility at Sanford Burnham at Lake Nona, Orlando Florida. Illumina's Truseq ChIP Library Preparation kit was used to prepare a total of 15 libraries (5 fragments each for 3 samples: uncut, sorted Dim, and sorted Bright) from 10ng of input DNA. Quality and quantity of the libraries were analyzed using an Agilent Bioanalyzer and Kapa Biosystems qPCR. The Multiplexed libraries were pooled and subjected to Paired-end 2×250-bp sequencing using one flow-cell of a Miseq sequencing instrument.

### Methylation analysis

FastQ files were subjected to quality check using FASTQC software (https://www.bioinformatics.babraham.ac.uk/projects/fastqc/). Then, paired-end reads from the sequencer platform were merged together using PEAR tool [66] with a minimum of 40 overlapping residues as threshold (mean PHREAD score of at least 33) and merged FastQ files were converted in Fasta using Prinseq. [67] To analyze the methylation status of each amplicon, we used AMPLIMETHPROFILER [68] specifically designed for deep targeted bisulfite amplicon sequencing of multiple genomic regions. This pipeline is freely available at https://sourceforge.net/projects/amplimethprofiler and is organized as follows: first, it recognizes corresponding target region discarding PCR artifacts and reads that do not match expected lengths; then, reads are aligned to the corresponding bisulfite-converted reference using BLASTn [69]. We used very stringent parameters: fragment length threshold, 50%; threshold alignment primers, 80%; bisulfite conversion efficiency, 99% and threshold alignment to reference, 50%. The pipeline output format reports the methylation status for each CpG dinucleotide coded 0 as non-methylated, 1 as methylated, and 2 if the methylation state cannot be assessed. We use this output to perform the analysis. Quantitative methylation average for each site is represented by the ratio between the number of non-converted bases at that site and the total number of mapped reads. The abundance of each of the 2NCpG distinct epialleles (where NCpG stands for the number of CpG sites in the analyzed region) was evaluated for each sample by counting the number of passing filter reads containing that epiallele. Qualitative methylation analysis was performed using Qiime [70], which includes: 1. a “summary”, the number of profiles present in each input sample; 2. a “taxa_summary_plots”, the information on the distribution of methylation profile classes; 3. “alpha diversity”, the five alpha diversity metrics for each sample: a. number of different methylation profiles in the sample; b. shannon entropy; c. simpson index; d. Chao 1 index; e. number of singletons. Such metrics were computed through a rarefaction procedure to take into account biases derived from variable sequencing depth and; 4. “beta Diversity”, the distance between samples in terms of composition of their methylation profiles, measured by Bray–Curtis dissimilarity: 5. PCoA Principal Coordinates analysis.

## SUPPLEMENTARY MOVIE AND TABLES






