# Supplementary material for: Non-homologous end joining induced alterations in DNA methylation: A source of permanent epigenetic change
Source: Oncotarget. 2017 Mar 11;8(25):40359–72. doi: 10.18632/oncotarget.16122 (PMC5522286; doi:10.18632/oncotarget.16122)
Supplement: Supplementary file 1 [file oncotarget-08-40359-s001.pdf]

Non-homologous end joining induced alterations in DNA methylation: A source of permanent epigenetic change

SUPPLEMENTARY MOVIE AND TABLE

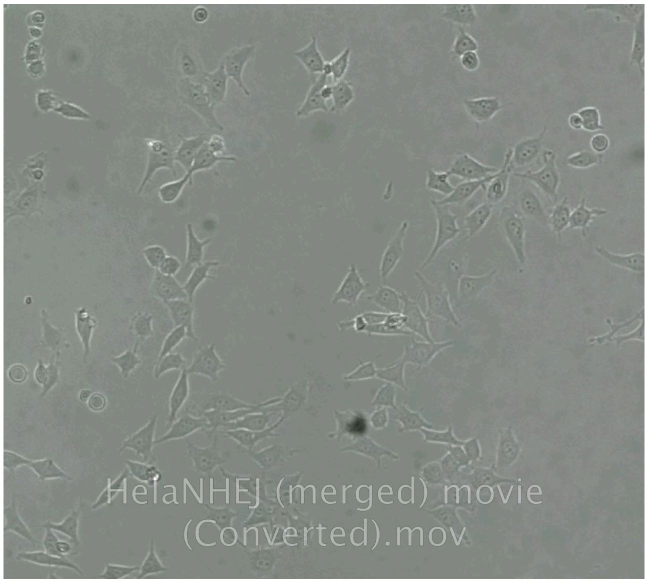

Supplementary Movie 1: Live cell imaging of dox-induced IHN20.22 cells.

See Supplementary Movie 1

Supplementary Table 1: Summary table of methylated profiles for region

See Supplementary File 1
