# Supplementary material for: Non-homologous end joining induced alterations in DNA methylation: A source of permanent epigenetic change
Source: Oncotarget. 2017 Mar 11;8(25):40359–72. doi: 10.18632/oncotarget.16122 (PMC5522286; doi:10.18632/oncotarget.16122)
Supplement: Supplementary file 2 [file oncotarget-08-40359-s002.docx]

Table S

Region 1

profiles	n_meths	UNCUT	DIMS	BRIGHT
○○	0	29.04	29.88	45.13
○●	1	59.78	56.10	47.77
●○	1	1.59	1.70	1.47
●●	2	9.57	12.30	5.61

Region 2

profiles	n_meths	UNCUT	DIMS	BRIGHT
○○○	0	14.64	5.85	44.90
○○●	1	12.38	17.59	9.12
○●○	1	0.76	1.02	0.66
●○○	1	0.56	0.81	1.54
○●●	2	9.45	9.57	1.86
●○●	2	5.52	7.83	2.44
●●○	2	3.80	3.27	5.82
●●●	3	52.84	54.01	33.63

Region 3

profiles	n_meths	UNCUT	DIMS	BRIGHT
○○	0	38.61	42.70	50.67
○●	1	16.01	16.07	6.85
●○	1	13.67	13.85	7.14
●●	2	31.69	27.36	35.32

Region 4

profiles	n_meths	UNCUT	DIMS	BRIGHT
○○○	0	9.14	16.01	37.60
○○●	1	2.22	3.77	3.55
○●○	1	2.20	3.04	2.23
●○○	1	3.79	6.37	6.91
○●●	2	17.86	17.88	12.04
●○●	2	2.76	3.12	1.83
●●○	2	4.60	4.86	2.95
●●●	3	57.39	44.90	32.85

Region 5

profiles	n_meths	UNCUT	DIMS	BRIGHT
○○○○	0	24.15	12.45	40.45
○○○●	1	2.86	1.52	4.194
○○●○	1	0	0	0.02
○●○○	1	5.76	6.21	5.54
●○○○	1	10.68	14.05	7.93
○○●●	2	0	0	0
○●○●	2	0.91	1	0.76
○●●○	2	0	0	0
●○○●	2	1.96	2.95	1.26
●○●○	2	0	0	0
●●○○	2	45.06	51.75	33.58
○●●●	3	0	0	0
●○●●	3	0	0	0
●●○●	3	8.56	9.96	6.18
●●●○	3	0	0.03	0.01
●●●●	4	0	0.02	0
